# Protective effect of miR‐18a in resected liver metastases of colorectal cancer and FOLFOX treatment

**DOI:** 10.1002/cnr2.1899

**Published:** 2023-09-12

**Authors:** Clemens Franz, Laila Jötten, Michael Wührl, Sibylle Hartmann, Fee Klupp, Thomas Schmidt, Martin Schneider

**Affiliations:** ^1^ Department of General, Visceral and Transplant Surgery University Hospital Heidelberg Heidelberg Germany

**Keywords:** colorectal cancer, FOLFOX protocol, hepatectomy, MicroRNAs, neoplasm metastasis

## Abstract

**Background:**

Colorectal cancer ranks second in terms of cancer associated deaths worldwide, whereas miRNA play a pivotal role in the etiology of cancer and its metastases.

**Aims:**

Studying the expression and cellular function of miR‐18a in metastatic colorectal cancer and association to progression‐free survival.

**Methods and Results:**

Colorectal liver metastases (*N* = 123) and primary colorectal cancer (*N* = 27) where analyzed by RT‐PCR and correlated with clinical follow up data. Invasion and migration assays were performed with the liver metastatic cell line LIM2099 after miR‐18a knockdown. Cell viability under FOLFOX treatment and knockdown was measured. We found that the expression of miR‐18a was increased 4.38‐fold in liver metastases and 3.86‐fold in colorectal tumor tissue compared to healthy liver tissue and colorectal mucosa, respectively (*p* ≤ .001). Patients with a high miR‐18a expression in liver metastases had a progression‐free survival (PFS) of 13.6 months versus 8.9 months in patients with low expression (*N* = 123; *p* = .024). In vitro migration of LIM2099 cells was reduced after miR‐18a knockdown and cell viability was significantly increased after miR‐18a knockdown and treatment with folinic acid or oxaliplatin. Subgroup analysis of PFS revealed significant benefits for patients with high miR‐18a expression receiving 5‐FU, folinic acid or oxaliplatin.

**Conclusions:**

High expression of miR‐18a in colorectal liver metastases might have a protective effect after resection of metastases and FOLFOX treatment regarding PFS.

## 1 INTRODUCTION

The epigenetic phenotype of cancer cells has become an aspect of upcoming importance in cancer and a molecular hallmark for diagnostic and treatment.^1^ MiRNAs can be oncogenic, tumor suppressive or regulatory, and play a pivotal role in etiology of colorectal cancer (CRC).^2^ With an incidence of about 1.9 million cases per year, CRC is the third most common cancer worldwide after breast and lung cancer. In terms of mortality, CRC is even second on the list with more than 935 000 deaths per year, and thus currently has the second highest mortality of cancers worldwide after lung cancer.^3^, ^4^, ^5^ Synchronous or metachronous metastases develop in approximately 20% of cases, which is associated with a median survival of 1.4 years.^6^ Identification of relevant oncogenes and tumor suppressors that contribute or inhibit carcinogenesis and metastasis formation is crucial for a better understanding of CRC and to apply and improve individualized targeted therapies. In a multimodal and tailored treatment concept, radiation and systemic treatment play an important role in addition to surgical therapy. Both neoadjuvant and adjuvant therapies can be considered for this purpose. In this context, the FOLFOX regimen is one of the most commonly used and usually recommended chemotherapy procedures for CRC from stage III.^7^, ^8^, ^9^, ^10^, ^11^

MiRNAs are small, non‐coding RNA molecules with a length of about 22 (18–25) nucleotides. Mature miRNAs bind to mRNA in the cytoplasm and can thereby prevent their translation or lead to their degradation.^1^, ^12^ It has been shown that more than one‐third of the human genome is regulated by miRNAs.^13^ Numerous studies have observed that a wide variety of tumor diseases are associated with alterations in the expression of miRNAs.^14^, ^15^, ^16^, ^17^, ^18^, ^19^ MiRNAs act as oncogenes or tumor suppressor genes, among others, and may also influence cancer therapy.^15^, ^18^, ^20^, ^21^, ^22^, ^23^, ^24^, ^25^, ^26^, ^27^ Thereby miRNAs play an upcoming role in progression of CRC and its liver metastasis by alternation of Wnt/β‐catenin, EGFR, TGF‐β, and TP53 pathway.^28^

MiR‐18a is part of the miRNA‐17‐92 cluster, which is one of the most studied clusters and is already known for its oncogenic effects and overexpression in a wide variety of cancers.^20^, ^29^, ^30^, ^31^, ^32^ In contrast, the exact function of each member of the cluster appears to be relatively unexplored. For miR‐18a, some studies have already shown overexpression in a wide variety of cancers, including CRC.^33^, ^34^, ^35^, ^36^ However, the role in carcinogenesis and whether miR‐18a acts more as an oncogene or tumor suppressor in CRC liver metastases in this context seems to be largely unresolved so far and opposing results are published.^37^, ^38^ With regard to a possible influence of miR‐18a expression on cancer therapy, only isolated studies exist. For instance, miR‐18a has a positive effect on radiation sensitivity in cervical cancer.^39^, ^40^ Improved antibody (trastuzumab) efficacy in breast cancer has also been demonstrated.^41^ In colorectal cancer, there is also preliminary evidence of enhanced chemosensitivity to 5‐FU and OX, which is associated with overexpression of miR‐18a.^42^ On the other hand, an opposite effect of miR‐18a was described and an overexpression was associated with an increased chemoresistance.^43^, ^44^, ^45^ Studying the expression and cellular function of miR‐18a could be helpful to gain a better understanding regarding the development, progression, and prognosis of mCRC and might help to apply this knowledge for the development of individualized targeted therapy.

## 2 MATERIALS AND METHODS

### 2.1 Patient samples

All tissue samples used, originate from the tissue bank for basic research and a clinical database with retro‐ and prospective data collection for clinical research and quality assurance in patients with diseases of the gastrointestinal tract and endocrine organs at Heidelberg University Hospital. Tissue samples were collected during surgeries between 2004 and 2015. All patients whose tissue was used had liver metastatic colorectal carcinoma with either simultaneously or staged resection of primary tumor and liver metastases. Informed consent was given by all patients. Inclusion in the analysis was conditional on the presence of both healthy colorectal mucosa, colorectal tumor tissue, healthy liver tissue, and tissue from a hepatic metastasis of colorectal carcinoma. Flash frozen tissue was sliced to a thickness of 8 μm using a CM 3050 S Cryostat microtome (Leica) and hematoxylin‐eosin staining was performed as verification of correct classification to the respective compartments. Clinical characteristics of patients are shown in Table 1.

**TABLE 1:** Samples of CRC liver metastases after liver resection of 123 patients were analyzed.

Patient characteristics	*N* = 123	Percentage
Gender		
Male	70	57%
Female	53	43%
Primary tumor		
Colon	76	62%
Rectum	47	38%
Colorectal liver metastases		
Synchron	81	66%
Metachron	42	34%
Liver resection		
Atypical	36	29%
Segmental	40	33%
Left hemihepatektomie	8	7%
Right hemihepatectomy	39	32%
CTx treatment		
Yes	106	86%
5‐FU	101	82%
Oxaliplatin	76	77%
Folinic acid	95	62%
No CTx	17	14%

### 2.2 Cell culture

The cell line LIM2099 (ECACC 12062002) from Dr. Robert Whitehead (Ludwig Institute for Cancer Research, Melbourne, Australia), derived from a human liver metastasis of colorectal adenocarcinoma, was used for all in vitro experiments. LIM2099 are MS‐stable, KRAS and beta‐catenin mutant cells, which express neither vimentin nor E‐cadherin. Histology shows molecularly differentiated epithelial cells with adherent growth.^46^, ^47^ RPMI‐1640 medium containing 25 mM HEPES (Sigma‐Aldrich Chemie GmbH, Steinheim, Germany), 10% FCS, 1% L‐glutamine, and 1% penicillin–streptomycin was used as culture medium. Additively, 7.85 IU human insulin (0.6 μg/mL), 550 μg hydrocortisone (1 μg/mL), and 4.4 μL 1‐thioglycerol (10 μM) were added to the medium. Thawing, culturing and splitting of cells was always performed under a class II biological safety cabinet (Thermo Electron LED GmbH, Langenselbold, Germany).

### 2.3 Real‐time PCR

Homogenization of tissue samples was performed using TissueLyser II (Qiagen GmbH, Hilden, Germany). RNA isolation of homogenized tissue or cell cultured cells was performed using the miRNeasy Mini Kit and reverse transcription using the miScript II RT Kits (each Qiagen GmbH, Hilden, Germany, CN 217004 and 218161). Forward primers for miR‐18a were used together with 10× miScript universal primer as the reverse primer. RNU6B was used as the endogenous control (each Qiagen GmbH, Hilden, Germany, CN MS00031514 and MS00033740). Semiquantitative RT‐PCR was set up using the GoTaq RT‐PCR Master Mix kits (Promega Corporation, Madison, WI, USA, CN A6002). Measurement was performed using LightCycler 480 II and analysis was performed using LightCycler® 480 software release 1.5.1.62 SP3 (each Roche Diagnostics Ltd., Rotkreuz, Switzerland, CN 27653). *C*_*t*_ values were calculated for each sample. According to the ΔΔ *C*_*t*_‐method as described by Livak and Schmittgen,^48^ RQ values were calculated as means of independent experiments and presented as expression fold changes.

### 2.4 Knockdown of miR‐18a

Anti‐hsa‐miR‐18a‐5p miScript miRNA inhibitor (Qiagen GmbH, Hilden, Germany, CN MIN0000072) was used for knockdown experiments. Transfection of the inhibitors into the cell was achieved using HiPerFect transfection reagent and miScript Inhibitor Negative Control (each Qiagen GmbH, Hilden, Germany, CN 301704 and 1027271) was used as control group instead of the inhibitor. 150 000 cells were seeded in 500 μL medium per well in a 24‐well plate. 0.3 μL of miRNA Inhibitor or Negative Control and 3 μL of HiPerFect transfection reagent were dissolved in 100 μL of RPMI‐1640 medium, mixed by vortexing briefly and added to the cells. The mixture was then incubated for 24 h at 37°C and 5% CO_2_.

### 2.5 Migration and invasion assay

For migration and invasion assays, ThinCerts™—TC inserts with a pore size of 8.0 μm (Greiner Bio‐One, Monroe, NC, USA, CN 662638) were added to a 24‐well plate. 100 μL of dissolved Corning® Matrigel® Basement Membrane Matrix (Corning Inc., Corning, NY, USA, CN 354234) was added to half of the inserts. 600 μL of nutrient‐depleted medium was added to the lower chamber, 100 000 cells were added to the upper chamber, and the plate was incubated for 18 h at 37°C and 5% CO_2_. After 18 h, the lower chamber medium was replaced with 600 μL of chemotactic nutrient‐rich medium, and incubated again for 24 h. The plate was then removed from the lower chamber. The upper chamber was then removed, carefully washed and cleaned from the inside. The inserts were placed in 600 μL crystal violet and stained for 10 min on a rocking shaker. The inserts were washed again and incubated in 600 μL of 10% acetic acid for 10 min. In each case, 100 μL of the cell‐containing acetic acid was added to one well of a 96‐well plate and the absorbance was determined photometrically at 540 nm and the mean was calculated.

### 2.6 Analysis of miR‐18a expression on PFS

miR‐18a expression levels in tissues of liver metastases in patients with mCRC were analyzed using RT‐PCR. Patients with an expression lower than the median were compared to patients with an expression that was equal or higher than the median. PFS was determined by retrospect analysis of clinical follow‐up routine investigations. Subgroup analysis of patients receiving chemotherapeutic treatment or not were performed. Specific reference was made to whether or not patients received 5‐FU, FOL, and OX (FOLFOX).

### 2.7 Chemotherapy assay in vitro

Following knockdown, 10 000 cells per well suspended in 100 μL culture medium were seeded onto a 96‐well plate. Cells were then treated with either 100 μM 5‐FU, 200 μM FOL, or 100 μM OX and incubated at 37°C and 5% CO_2_ for 48 h. Cell proliferation and viability was evaluated using a WST‐1 assay. 10 μL of Cell Proliferation Reagent WST‐1 (Sigma‐Aldrich Chemie GmbH, Steinheim, Germany, CN 11644807001) was added to each well and the plates were incubated for 1 h at 37°C and fluorescence was measured photometrically at 450 nm versus 630 nm.

### 2.8 Statistical analysis

Statistical analysis was performed using IBM® SPSS® Statistics Software (Release 28). Metric variables like expression, cell proliferation and cell survival were tested for normal distribution with Shapiro–Wilks‐test for normal distribution. Comparison of two groups with not normally distributed data was performed with the Wilcoxon rank sum test. For normally distributed data, the Student's *t*‐test was applied. For comparison of more than two groups, the ANOVA test with Tukey's post‐hoc test was used. Survival analysis for progression‐free survival (PFS) were performed with the Kaplan–Meier method. Differences in survival between subgroups were analyzed with the log‐rank test (Mantel‐Cox). For all tests, the significance level was set at *p* ≤ .05.

## 3 RESULTS

### 3.1 Expression of miR‐18a in metastatic colorectal cancer

**FIGURE 1:**
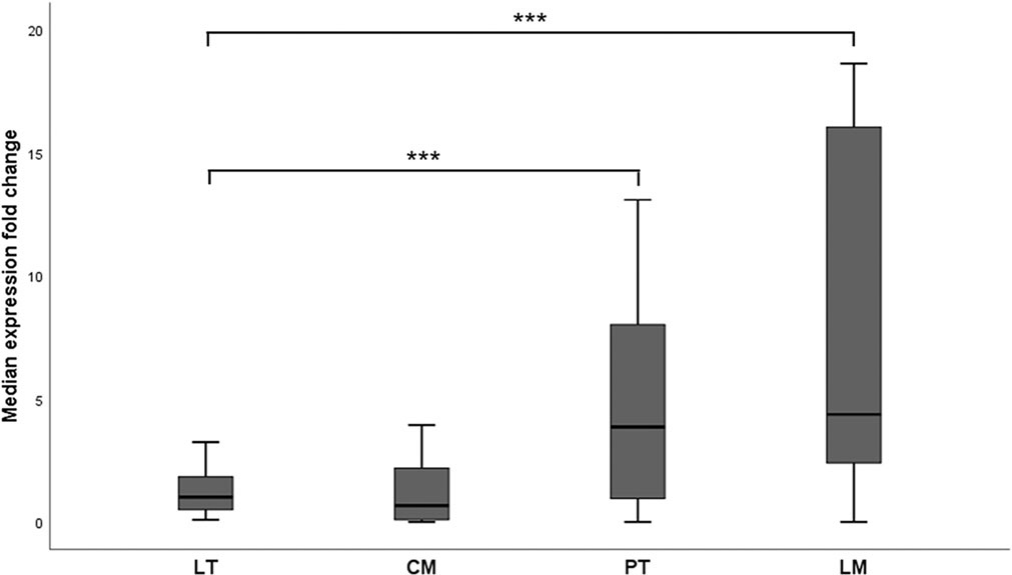
Expression of miR‐18a in different tissue compartments of patients with mCRC. RT‐PCR showing the miR‐18a expression fold change in patients with mCRC. Four different tissue compartments were examined: LT, CM, PT, and LM. PT and LM revealed a significantly higher expression of miR‐18a compared to LT and CM. *N* = 27, ***p ≤ .001. CM, colorectal mucosa; LM, liver metastasis; LT, liver tissue; PT, primary tumor.

The expression of miR‐18a in four different tissue compartments of mCRC was analyzed by RT‐PCR. Healthy liver tissue was taken as control, since the focus was on CRC liver metastases, whereas surgery for liver metastases and primary tumor was mostly performed at two different time points. A significantly increased expression of miR‐18a was found in colorectal tumor tissue (median: 3.86; *p* = .001; Figure 1) as well as in liver metastases (median: 4.38; *p* ≤ .001; Figure 1) compared to healthy liver tissue. In contrast, colorectal mucosa showed the lowest miR‐18a expression but did not differ significantly from healthy liver tissue (median: 0.66; *p* = .570; Figure 1). The patient characteristics listed in Table 1 showed overall nonsignificant *p*‐values with respect to a difference in miR‐18a expression.

### 3.2 Influence of miR‐18a expression on PFS

**FIGURE 2:**
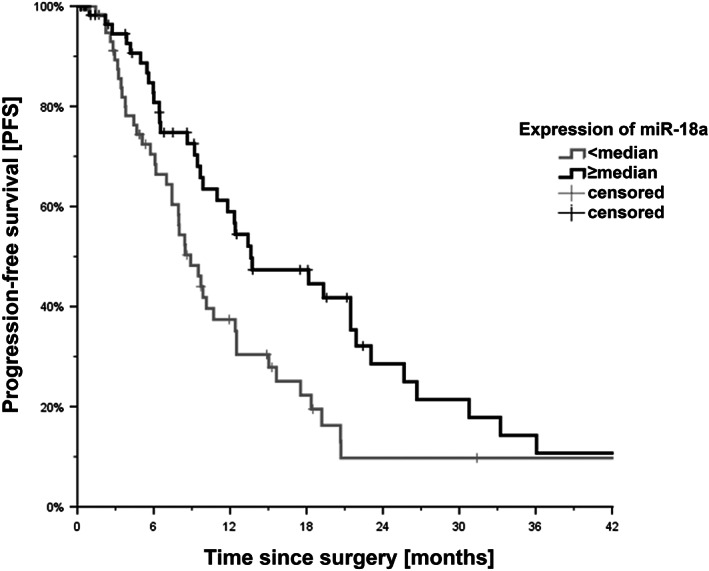
Impact of miR‐18a expression on PFS after resection of liver metastases. Kaplan–Meier curves revealing PFS in months after resection of CRC liver metastases. Patients with a lower expression of miR‐18a than the median showed an impaired PFS. Expression of miR‐18a higher than the median resulted in a prolonged PFS. *N* = 123, *p* = .024. miR‐18a, microRNA‐18a; PFS, progression‐free survival.

To analyze the influence of miR‐18a on PFS, the results of RT‐PCR were evaluated in conjunction with the respective patient data. Despite the mean follow up time of 27.5 months and patients included within the study that undergo liver resection and multimodal therapy, the occurrence of the event “death” numerically not high. Therefore, no valid statistical analysis was performed in this regard, which was also not the aim of the work. Median PFS after liver resection of metastases was 11.0 months (95% CI, 8.5–13.4; Figure 2). Patients with a low miR‐18a expression had a PFS of 8.9 months (95% CI, 7.0–10.8; Figure 2), whereas patients with an increased expression of miR‐18a showed a significantly prolonged PFS of 13.6 months (95% CI, 6.7–20.6; *N* = 123; *p* = .024; Figure 2).

### 3.3 Migration and invasion after in vitro miR‐18a knockdown

**FIGURE 3:**
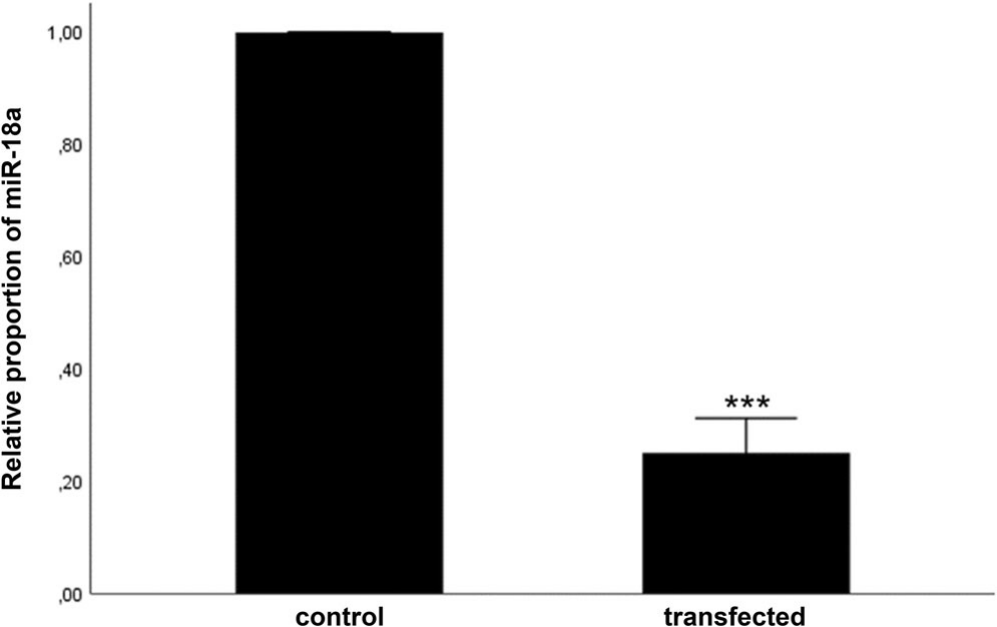
Targeted knockdown of miR‐18a in LIM2099. RT‐PCR showing the altered expression of miR‐18a in cell culture (CRC liver metastatic cell line LIM2099) after targeted knockdown of miR‐18a by transfection. The bar graph shows the results of RQ values of RT‐PCR as the mean value and its standard error. *N* = 5; ***p ≤ .001. RT‐PCR, real‐time polymerase chain reaction; RQ, relative quotient.

**FIGURE 4:**
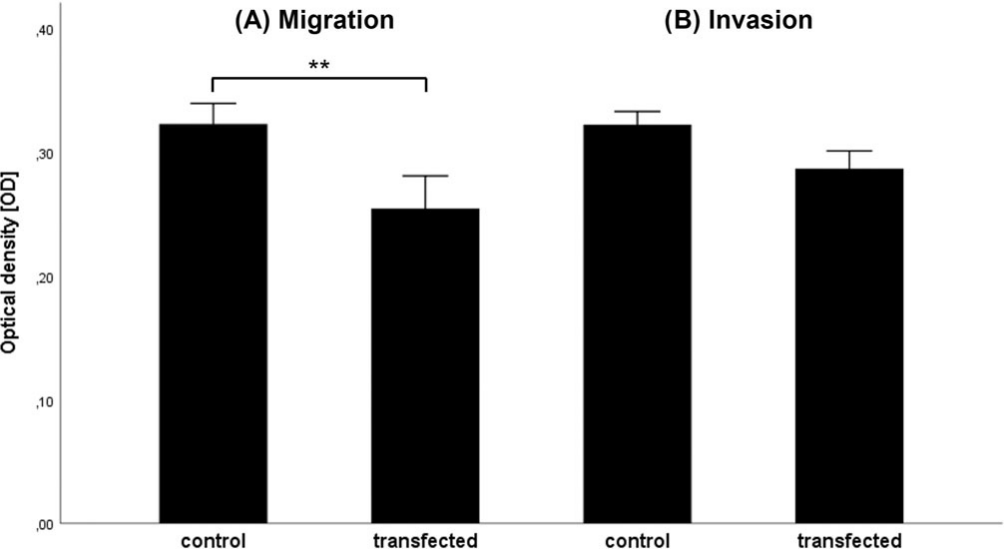
Migration and invasion assay. In vitro migration and invasion assays revealing the migratory (A) or invasive (B) potential of LIM2099. Transfected cells with humiliated miR‐18a expression were compared to control group. The mean OD of the photometric measurement was plotted with their standard error. (A) Migration assay. *N* = 14; **p = .004. (B) Invasion assay. *N* = 15; *p* = .105. OD, optical density.

Migration and invasion assays were performed with LIM2099 cells after knockdown of miR‐18a as described above. Overall knockdown efficiency was 75% (0.7493; Figure 3). After knockdown of miR‐18a, a significant reduction of cell migration of about 23% was found compared to control group (Figure 4A). Invasion of cells after miR‐18a knockdown showed a nonsignificant reduction of 9% compared to the control group (Figure 4B). Growth controls did not show a significantly different proliferation rate of cells after knockdown compared to the control group.

### 3.4 miR‐18a expression and PFS under FOLFOX‐treatment in vivo

**FIGURE 5:**
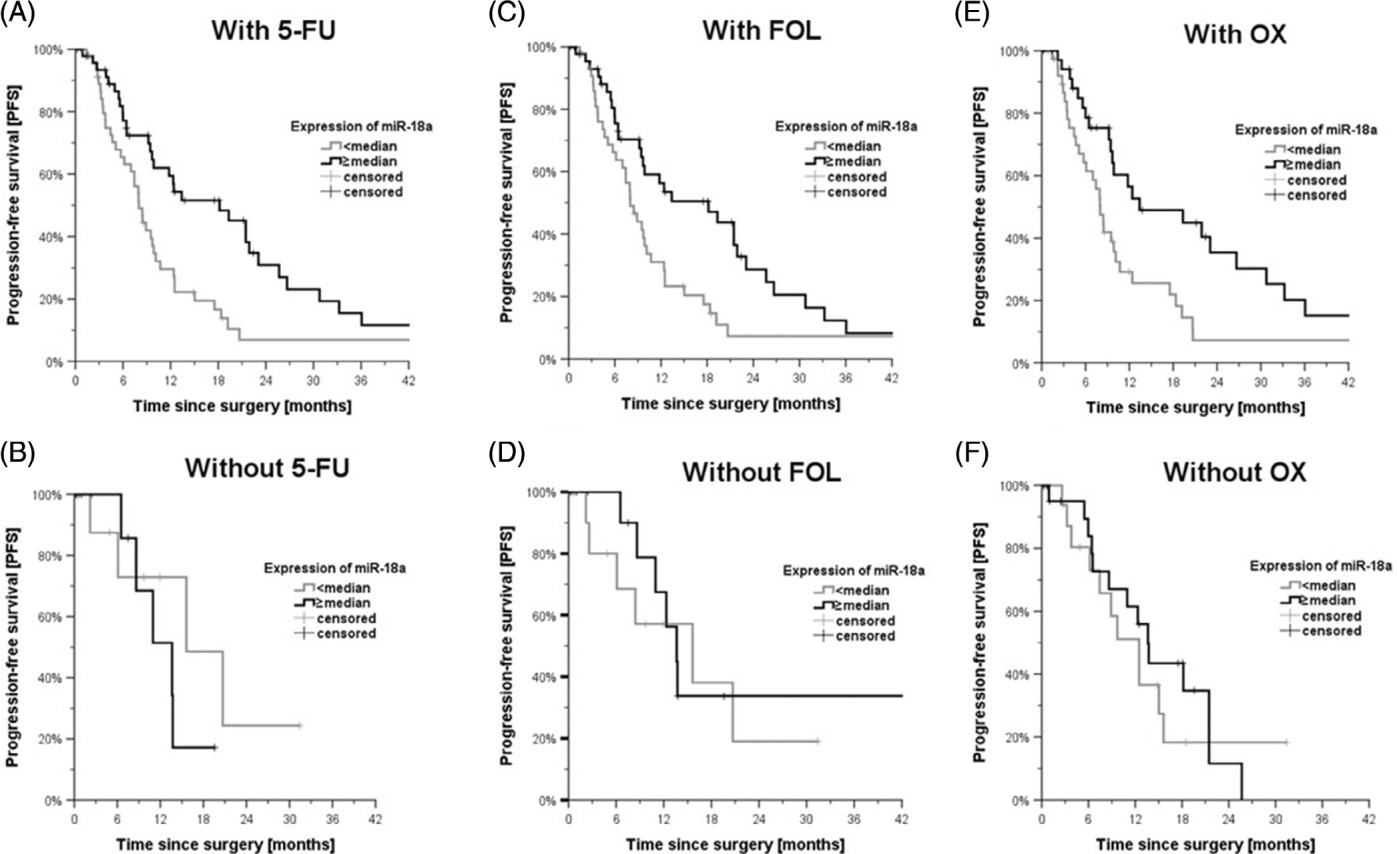
Influence of miR‐18a on PFS under FOLFOX‐treatment in vivo. Kaplan–Meier curves showing PFS in months after resection of CRC liver metastases. Patients with a lower expression of miR‐18a than the median were compared to patients with higher expression than the median. (A) PFS in patients with high or low miR‐18a expression that received treatment with 5‐FU. *N* = 96; *p* = .005. (B) In contrast, patients with high or low miR‐18a expression without 5‐FU treatment. *N* = 20; *p* = .375. (C) PFS in patients with high or low miR‐18a expression that received treatment with FOL. *N* = 90; *p* = .019. (D) In contrast, patients with high or low miR‐18a expression without FOL treatment. *N* = 26; *p* = .686. (E) PFS in patients with high or low miR‐18a expression that received treatment with OX. *N* = 76; *p* = .015. (F) In contrast, patients with high or low miR‐18a expression without OX treatment. *N* = 40; *p* = .615. 5‐FU, 5‐fluorouracil; FOL, folinic acid/leucovorin; FOLFOX, FOL/5‐FU/OX; miR‐18a, microRNA‐18a; OX, oxaliplatin; PFS, progression‐free survival.

With the aim of explaining the different PFS for high and low miR‐18a expression and following the in vitro results above, we hypothesized a differential expression of miR‐18a might have an impact on therapy sensitivity and thus on PFS. Therefore, subgroup analysis of patient data and miR‐18a expression was performed. Here, an impact of miR‐18a expression on FOLFOX therapy could be identified. Within the subgroup of patients receiving 5‐FU, PFS was significantly impaired with 8.0 months (95% CI 6.7–9.3) in patients with low expression levels of miR‐18a compared to 18.2 months (95% CI 10.5–25.9) for patients with high expression levels (Figure 5A). This effect was not observed in the group of patients not receiving 5‐FU (Figure 5B). For the group of patients who received treatment with FOL, a significantly improved PFS was observed as well with 8.0 months (95% CI 62–9.8) in patients with low expression levels of miR‐18a compared to 18.2 months (95% CI 8.4–28.0) in patients with high expression levels (Figure 5C). In contrast, this effect was not observed in the group of patients not treated with 5‐FU (Figure 5D). Patients receiving OX had a significantly impaired PFS with 8.0 months (95% CI 6.5–9.5) in patients with low expression levels of miR‐18a compared to 13.4 months (95% CI 1.4–25.4) in patients with high expression levels (Figure 5E). Again, this difference was not observed in the group of patients without 5‐FU therapy (Figure 5F).

### 3.5 Influence of miR‐18a on tumor cell survival under FOLFOX‐treatment in vitro

**FIGURE 6:**
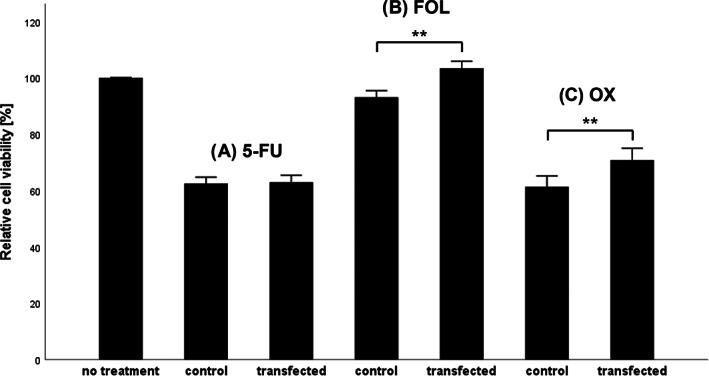
Influence of miR‐18a on LIM2099 cell proliferation and survival under FOLFOX‐treatment in vitro. WST‐1 assay of LIM2099 cells after 48 h of treatment with FOLFOX substances for miR‐18a knockdown and control cells. Control and transfection groups are shown separately with mean values and its standard error. (A) Cell survival after treatment with 5‐FU. *N* = 7; *p* = .881. (B) Cell survival after treatment with FOL. *N* = 7; *p* = .008. (C) Cell survival after treatment with OX. *N* = 7; *p* = .010. 5‐FU, 5‐fluorouracil; FOL, folinic acid/leucovorin; FOLFOX, FOL/5‐FU/OX; miR‐18a, microRNA‐18a; OX, oxaliplatin; WST‐1, water‐soluble tetrazolium salt 1. **p = .004.

For further investigation of these findings, additional in vitro experiments with the three therapeutics were subsequently performed with the mCRC cell line LIM2099. Therefore, the influence on proliferation and cell survival with high and low miR‐18a expression for each separate drug 5‐FU, OX and FOL was analyzed in vitro with specific miR‐18a knockdown. Under treatment with 5‐FU, no significant difference in cell survival was found when analyzing transfected cells with low miR‐18a expression compared to the control group with high miR‐18a expression (Figure 6A). In contrast, under treatment with FOL the cell survival in the transfection group with low miR‐18a expression was significantly higher than in the control group with high miR‐18a expression (103% and 93%, respectively, *p* = .008; Figure 6B). Similar observations could be made for OX, where transfected cells with low miR‐18a expression showed significantly improved cell survival under OX treatment compared to the control group (71% vs. 61%, respectively, *p* = .01; Figure 6C).

## 4 DISCUSSION

Analysis of cellular mechanisms with the identification of over‐ and under expression of RNAs in various tumors is crucial for a more detailed understanding of the oncologic diseases. miRNAs are detectable in all biological fluids and could be useful for individualized treatment within screening, diagnosis, follow‐up, and prognosis.^49^, ^50^ Numerous studies have already demonstrated overexpression of miR‐18a in the primary tumor of colorectal carcinomas.^34^, ^36^, ^51^ With this study, we can confirm the overexpression of miR‐18a in the primary tumor of CRC and furthermore we could prove its overexpression in CRC liver metastases (Figure 1).

While the increased expression of miR‐18a in colorectal carcinomas has been shown and reproduced several times, results in the literature differ regarding the impact of overexpression on the disease. It is currently discussed whether miR‐18a is an oncogene or a tumor suppressor and to what extent it influences survival. Slattery et al.^52^ showed that miR‐18a significantly reduced the risk of death. Another work by Yu et al.^42^ demonstrated that decreased expression of miR‐18a was associated with increased chemoresistance in vivo and in vitro. In our work, we could demonstrate that increased expression of miR‐18a in liver metastases of CRC was associated with significantly prolonged PFS for patients with mCRC (Figure 2). Further studies seem necessary to delineate the cellular mechanisms of this effect.

In the literature, the miR‐17‐92 cluster is already known to be an oncogene in a wide variety of tumor types.^20^, ^30^, ^31^ However, it remains unclear what role each member of the cluster plays in tumorigenesis. For miR‐18a, both an oncogenic and tumor suppressive function is discussed.^34^, ^36^, ^53^, ^54^, ^55^, ^56^, ^57^, ^58^, ^59^, ^60^, ^61^, ^62^, ^63^ In the migration and invasion assays on the mCRC cell line LIM2099, we aimed to investigate the effect of miR‐18a on tumor carcinogenesis. Here, significantly reduced cell migration was observed under suppression of miR‐18a (Figure 4). Invasion again showed a nonsignificant difference but at least a tendency to reduced invasion under miR‐18a knockdown (Figure 4). These results support the hypothesis of an oncogenic effect of miR‐18a. Regarding the existing discrepancy in the literature, theories exist that cells may have distinct miRNA profiles depending on their developmental stage.^64^ Thus, the function of individual miRNAs might depend on tumor entity, stage, and individual cellular differences.^36^ In addition, a dual mode of action of individual miRNAs is discussed, in which they take on both oncogenic and tumor suppressive functions. Evidence already exists for the miR‐17‐92 cluster regarding such a dual role and further investigations should follow.^34^, ^65^

Based on the above results, it was hypothesized that in the absence of a tumor suppressive effect of miR‐18a in cell culture, the significantly prolonged PFS upon overexpression might be due to a change in therapy sensitivity. While mRNA expression has been shown to influence cancer therapy in numerous ways,^15^, ^18^, ^24^ there is currently only one paper showing that increased expression of miR‐18a in the primary tumor is associated with significantly prolonged PFS in CRC.^42^ In another study, miR‐18a was observed to have a positive effect on the risk of death in CRC.^52^ Within this study, we demonstrated that the significantly prolonged PFS in mCRC was coherent with the expression of miR‐18a on therapy with FOLFOX (Figure 5). Thus, the observed effect did not appear to be present within the subgroup of patients without either of the corresponding therapeutic agents. These results are limited by the lack of discrimination with respect to the individual agents of FOLFOX, as they are usually administered together within the treatment regimen. Considering these limitations, it seemed reasonable to follow up with further experiments within the cell culture. Here, both FOL and OX showed significantly improved cell survival of LIM2099 under suppression of miR‐18a by targeted knockdown (Figure 6). These results support the theory that increased expression of miR‐18a has a positive effect on mCRC therapy. Interestingly, this effect was not shown for 5‐FU. Unexpectedly, FOL alone in combination with high expression of miR‐18a showed its own cell toxic effect. This effect was explained in the literature with an antiproliferative, cell‐toxic activity of FOL, for example by inhibition of thymidylate synthase.^66^, ^67^ Wang et al. demonstrated a cytotoxic effect of FOL on CRC tumor growth in rats, but this effect was not significant.^68^ It is possible that this observation is dependent on the composition of FOL, which is administered in medicine as a combination preparation consisting of the natural and unnatural diastereoisomer of FOL.^66^ It is also interesting to note that folates within the body are usually found in polyglutamated form (folylpolyglutamates), as they are stored and transported in this constitution.^69^ With regard to these oligoglutamate derivatives, some studies exist which have shown that they were able to inhibit diverse enzymes of folate metabolism, including thymidylate synthase.^69^, ^70^, ^71^ It is possible that this represents a natural feedback mechanism within folate metabolism, which is evident at high doses of FOL.

In conclusion, our study could show the increased expression levels of miR‐18a in colorectal liver metastases and primary CRC. Analysis of clinical follow up data after resection of CRC liver metastases with prolonged PFS in patients with high metastatic miR‐18a expression and altered therapy sensitivity of mCRC cells in vitro depending on miR‐18a expression seem interesting. In conjunction with the clinical relevance of mCRC, further studies in this regard are needed to identify the underlying intra‐ and extracellular mechanisms that cause this effect.

## AUTHOR CONTRIBUTIONS

**Clemens Franz:** Conceptualization (lead); data curation (equal); formal analysis (equal); funding acquisition (lead); investigation (equal); methodology (equal); project administration (lead); resources (equal); supervision (lead); validation (equal); visualization (equal); writing – original draft (lead); writing – review and editing (lead). **Laila Jötten:** Conceptualization (supporting); data curation (equal); formal analysis (equal); methodology (equal); resources (supporting); validation (supporting); visualization (equal); writing – original draft (supporting). **Michael Wührl:** Conceptualization (supporting); data curation (supporting); formal analysis (supporting); methodology (supporting); resources (supporting); validation (supporting); writing – original draft (supporting). **Sibylle Hartmann:** Conceptualization (supporting); data curation (supporting); formal analysis (supporting); methodology (supporting); resources (supporting); validation (supporting); writing – original draft (supporting). **Fee Klupp:** Conceptualization (supporting); data curation (supporting); formal analysis (supporting); investigation (supporting); resources (supporting); validation (supporting); writing – review and editing (supporting). **Thomas Schmidt:** Conceptualization (supporting); formal analysis (supporting); funding acquisition (supporting); investigation (supporting); resources (supporting); supervision (supporting); validation (supporting); writing – review and editing (supporting). **Martin Schneider:** Conceptualization (supporting); funding acquisition (supporting); investigation (supporting); project administration (supporting); resources (supporting); supervision (supporting); writing – review and editing (supporting).

## CONFLICT OF INTEREST STATEMENT

The authors have stated explicitly that there are no conflicts of interest in connection with this article.

## ETHICS STATEMENT

This project was approved by the Ethics Committee of the University of Heidelberg (Reference no. S‐649/2012 and S‐596/2015). Informed consent was obtained from all participants. The study was performed in accordance with the Declaration of Helsinki.

## Supporting information


**Figure S1:** Cell growth of LIM2099 in culture. Cell culture growth of LIM2099 (ECACC 12062002).

**Figure S2:** Mycoplasma test—Agarose gel electrophoresis. Agarose gel electrophoresis of a mycoplasma test; pc at 267 bp, nc as well as samples in the range of the internal control (191 bp), showing that the PCR has run successfully. nc, negative control; pc, positive control; s, sample.

**Figure S3:** Melting curve analysis for RNU6B and miR‐18a. Shown are three technical replicates each for RNU6B and miR‐18a and two technical replicates each of the negative controls of the primers (generated with LightCycler® 480 Software release 1.5.1.62 SP3, Roche Diagnostics). nc, negative control.

## Data Availability

Data of this study are available on request due to privacy/ethical restrictions.
