# Supplementary figures and images for: Protective effect of miR‐18a in resected liver metastases of colorectal cancer and FOLFOX treatment
Source: Cancer Rep (Hoboken). 2023 Sep 12;6(12):e1899. doi: 10.1002/cnr2.1899 (PMC10728504; doi:10.1002/cnr2.1899)

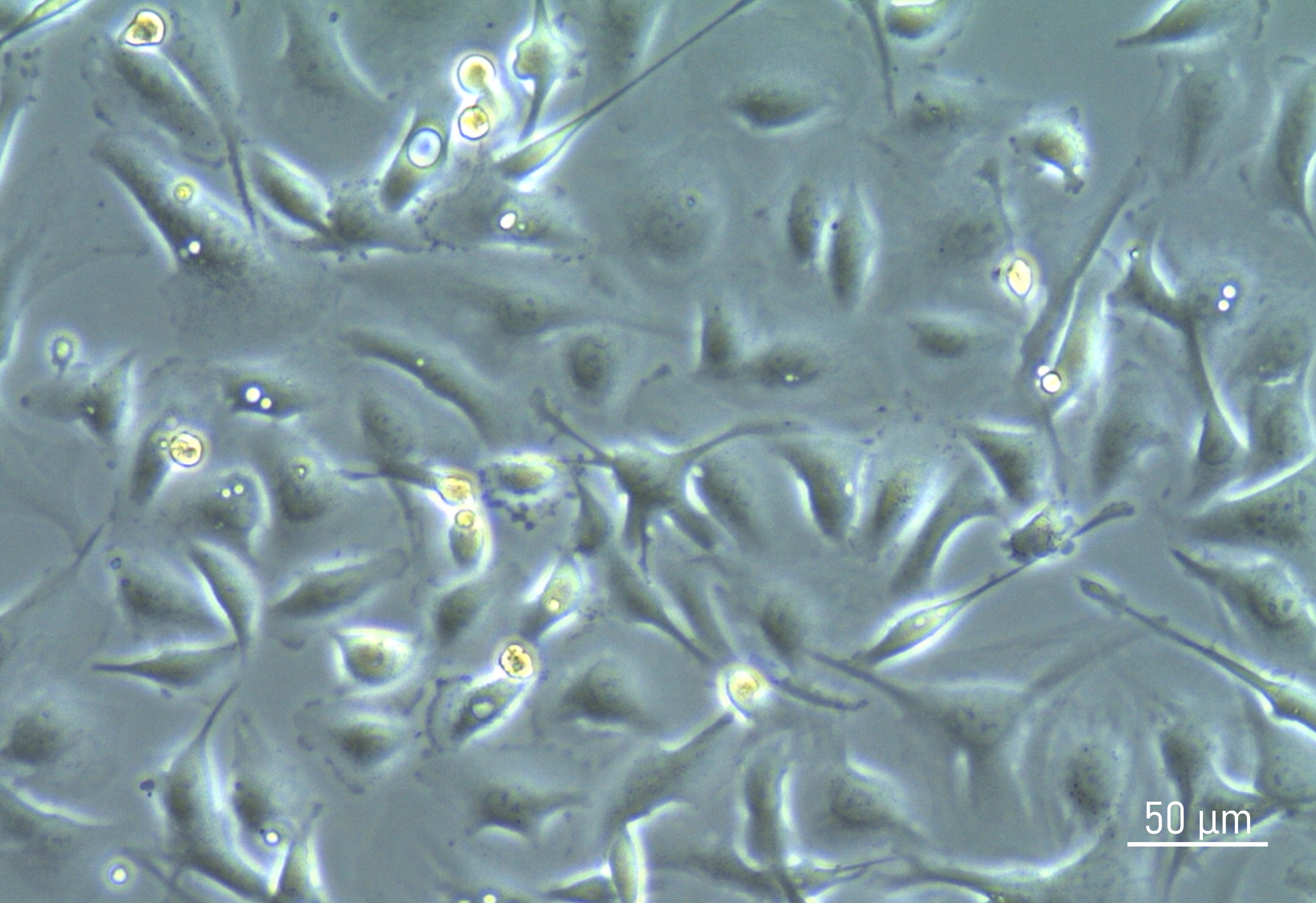

Supplement: Supplementary file 1 — Figure S1: Cell growth of LIM2099 in culture. Cell culture growth of LIM2099 (ECACC 12062002). [file CNR2-6-e1899-s001.jpg]

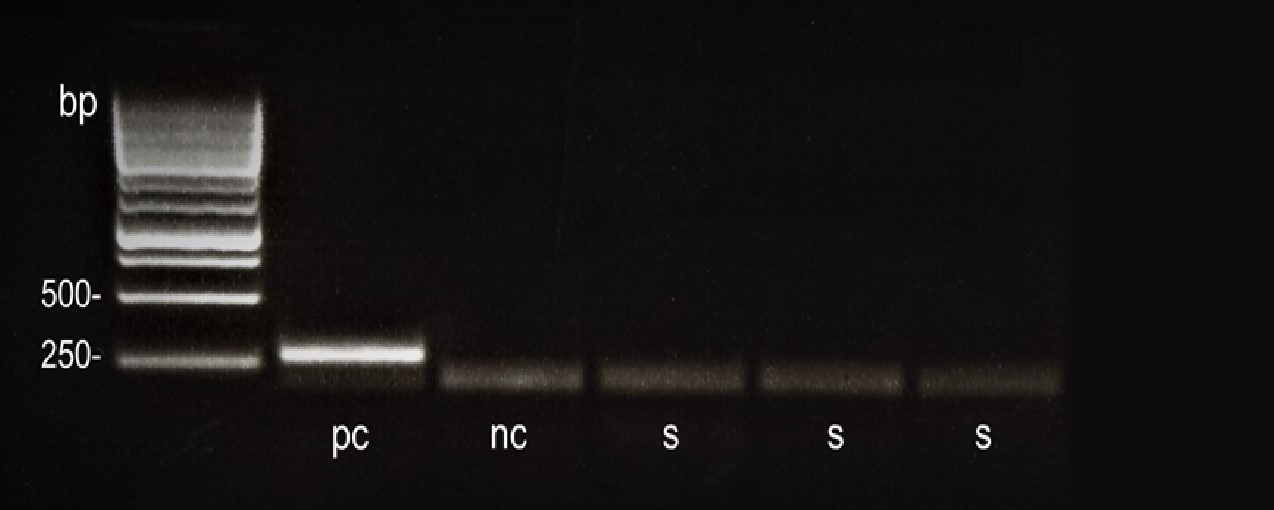

Supplement: Supplementary file 2 — Figure S2: Mycoplasma test—Agarose gel electrophoresis. Agarose gel electrophoresis of a mycoplasma test; pc at 267 bp, nc as well as samples in the range of the internal control (191 bp), showing that the PCR has run successfully. nc, negative control; pc, positive control; s, sample. [file CNR2-6-e1899-s002.jpg]

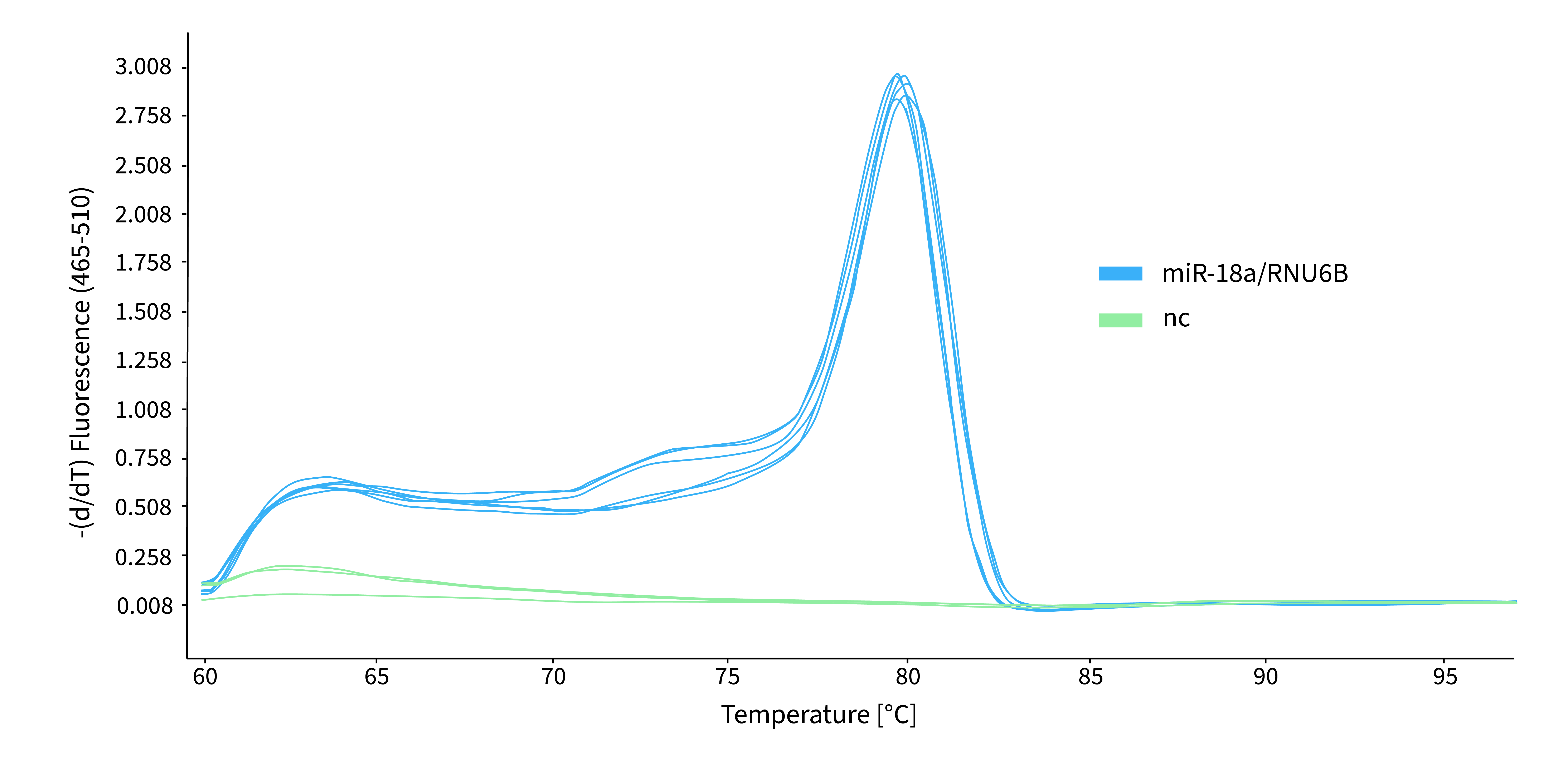

Supplement: Supplementary file 3 — Figure S3: Melting curve analysis for RNU6B and miR‐18a. Shown are three technical replicates each for RNU6B and miR‐18a and two technical replicates each of the negative controls of the primers (generated with LightCycler® 480 Software release 1.5.1.62 SP3, Roche Diagnostics). nc, negative control. [file CNR2-6-e1899-s003.jpg]
